# The Predictive Value of Computed Tomography Findings for Poor Visual Outcome in Traumatic Eye Injury

**DOI:** 10.1155/2022/4995185

**Published:** 2022-09-01

**Authors:** Ping Ren, Yan Jiao, Chunling Zhang, Guoyue Chen

**Affiliations:** Department of Radiology, Jinan Central Hospital, Jinan City, Shandong Province, 250013, China

## Abstract

**Background:**

The prognosis of visual outcome is important for patients and healthcare providers and guides proper decision-making in traumatic eye injury. In this study, we have evaluated the predictive value of computed tomography (CT) scan findings for poor visual outcomes in patients with traumatic eye injuries.

**Methods:**

In a retrospective survey, documents of 200 patients with traumatic eye injury who underwent a diagnostic orbital CT scan were reviewed. Disorganized or collapsed globe, intraocular foreign body or gas, increased or decreased anterior chamber size, hemorrhage in the anterior or posterior chamber, crystalline or intraocular lens dislocation, posterior sclera thickening, globe borders haziness, orbital fracture, orbital hemorrhage, and foreign body, optic canal, and optic nerve injuries are the diagnostic clues for eye injury in CT scan. The predictive value of CT scan findings for poor visual outcome was calculated by sensitivity, specificity, accuracy, predictive values, hazard ratios, and binary logistic regression model.

**Results:**

The sensitivity, specificity, accuracy, and positive predictive values showed to be high. However, there was a low negative predictive value of CT findings for the prediction of poor vision. Among the investigated factors, disorganized/collapsed globe (HR 47.72, CI 6.13–371.62), increased/decreased anterior chamber size (HR 5.04, CI 2.57–9.88), hemorrhage in anterior/posterior chamber (HR 3.58, CI 1.900–6.774/3.62, CI 1.90–6.89), globe borders haziness (HR 3.06, CI 1.33–7.01), orbital foreign body (HR 3.66, CI 1.11–12.05), and optic canal/nerve injury (HR 21.62, CI 4.73–98.78) reached the statistical significance for increasing the hazard ratio for poor visual outcome in patients with a traumatic eye injury. Logistic regression analysis showed only evidence for disorganized/collapsed globe and optic canal/nerve injury in orbital CT scan as independent predictive factors for poor visual outcome.

**Conclusion:**

CT scan findings can be used as prognostic factors for visual outcomes in patients with a traumatic eye injury.

## 1. Introduction

Traumatic eye injury is one of the important causes of blindness worldwide [1, 2]. The annual universal rate of traumatic eye injury is estimated to be 55 million cases, by the World Health Organization [3]. Young males are the most vulnerable population to traumatic eye injury, mostly by occupational trauma, which causes a significant lifelong burden of disease for the patients [4–7]. Vision loss is the most prevalent and important adverse outcome of traumatic eye injury. It is suggested that, annually, more than 22 million people lose their vision by trauma in the world [3]. Traumatic eye injury is considered the leading cause of unilateral vision loss which is reported to happen in more than 25% of cases affected by eye trauma [8].

Different prognostic factors are suggested for the prediction of visual outcomes in patients with traumatic eye injury [9–12]. Clinical findings such as presenting visual acuity, the presence of retinal lens and optic nerve injury, the site and the extent of the wound in open eye trauma, presence of hyphema, vitreous hemorrhage, and intraocular foreign body are suggested as predictive factors for final visual outcome in these patients [13, 14]. However, detailed evaluation of globe injury in trauma patients is challenging [15]. Poor cooperation of patients, low level of consciousness, and concurrent traumatic injuries to other parts limiting the ability of patients for slit-lamp examination limit the feasibility of accurate physical exam for the evaluation of a patient with eye trauma [16].

Imaging is popularly used for evaluating patients with eye trauma [17, 18]. Computed tomography (CT) scan is the most popular modality and imaging of the choice in these patients [19]. It has more diagnostic accuracy for periorbital bony and soft tissue injuries compared to radiography [20]. Unlike ultrasonography, a CT scan can be performed in suspected open globe injury [21] and has no limitation in patients with suspected intraocular or intraorbital foreign body which limits the use of magnetic resonance imaging [22]. CT scan does not need patient cooperation and can be used more easily in patients with trauma [18]. Different findings in CT scan have been suggested as the predictive factors associated for eye injury [18]. Disorganized or collapsed globe, intraocular foreign body or gas, increased or decreased anterior chamber size, hemorrhage in the anterior or posterior chamber, crystalline or intraocular lens dislocation, posterior sclera thickening, globe borders haziness, orbital fracture, orbital hemorrhage, and foreign body, optic canal, and optic nerve injuries are the diagnostic clues for eye injury in CT scan [23–25]. These factors can be used for the prediction of potential visual outcomes in patients presented with a traumatic eye injury. In this study, we have evaluated the predictive value of these findings for poor visual outcomes in patients with a traumatic eye injury.

## 2. Methods and Materials

### 2.1. Setting and Participants

The study protocol was approved by the local committee for ethics in research of our center (approval number: CT-2020-124). The records of patients referred to the academic referral center for ophthalmic emergencies, from January 2020 to December 2021 with the chief complaint of eye trauma and who underwent orbital CT scan, were reviewed for evaluation of eligibility criteria. Patients with the age of more than 18 years old of both gender were included in the studies. Records registered as trauma including blunt and penetrating injuries and excluding chemical injuries were enrolled. Records without arrival CT scans (for example, CT scans performed after surgical intervention) were excluded. Other exclusion criteria were lack of registration of follow-up best-corrected vision between 8 and 12 weeks of presentation and registered visual problem before the trauma. Poor visual outcome was defined as vision less than 20/1000 (equivalent to 1.7 LogMAR) based on previous definitions of near-total visual impairment [26].

### 2.2. CT Scan Technique and Interpretation

CT scans were taken by using multidetector CT scan devices (Aquilion 64, Toshiba Medical Systems) without any contrast agent with the thin cut protocol (2 mm cuts thickness) axial with coronal and sagittal reconstruction using 89–345 mA, 120 kV, 0.5–0.8 pitch, and 0.5–0.8 s rotation time. CT scan images were read by two independent expert radiologists without any information about clinical findings and the outcome of patients with a traumatic eye injury. In case of any disagreement, a third radiologist's opinion was applied.

### 2.3. Sample Size Calculation

Considering the potential 25% of final visual impairment in the patients presented with trauma and 50% positive findings in CT scan of those patients based on our preliminary evaluation and a one-sided alpha of 5% and a power of 80%, we reached the sample size of at least 200 patients for the study.

### 2.4. Statistical Analysis

Statistical analysis was performed with Statistical Package for the Social Sciences (IBM SPSS Statistics 26.0). All the descriptive data were presented as means and standard error of means for quantitative variables and frequency as well as percentages for qualitative variables. The predictive value of CT scan findings for poor visual outcome was calculated by sensitivity, specificity, accuracy, predictive values, hazard ratio, and binary logistic regression model. Cohen's kappa and percent of the agreement were used for evaluation of the interrater agreement [27]. The *p* value of less than 0.05 was considered statistically significant.

## 3. Results

### 3.1. Study Flow

**Figure 1:**
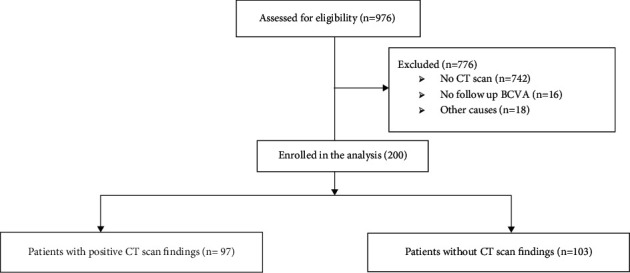
Flow diagram of the enrollment in the study.

Among 976 reviewed records of patients presented to the emergency department with a chief complaint of eye trauma, 200 records fulfilled the eligibility criteria for enrollment in the analysis. The details of the records excluded in each stage are presented in Figure 1.

### 3.2. Basic Characteristics

Most of the patients enrolled in the study (74.5%) were male. The mean age of the participants was 29.92 years old. Most of the patients were educated (79.5%). The most common settings of injury were home occurring (27%) and intentional (23%) injuries. There was no significant difference between patients with and without CT scan findings regarding their gender, age, educational status, and setting of trauma to the eye. The information on demographic characteristics in all enrolled patients and each study group are presented in detail in Table 1.

### 3.3. CT Findings

**Figure 2:**
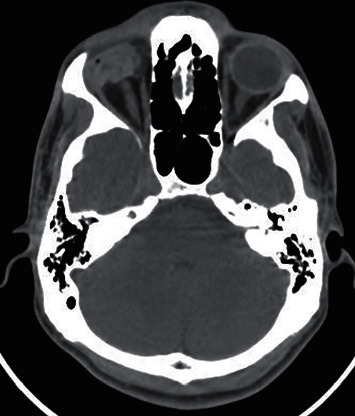
Open globe injury in a 26-year-old man. Axial unenhanced CT image showing right disorganized globe.

**Figure 3:**
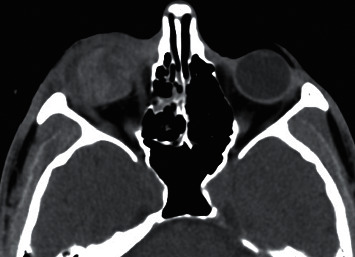
Open globe injury in a 53-year-old woman. Axial unenhanced CT image showing hemorrhage in posterior chamber of the right eye.

**Figure 4:**
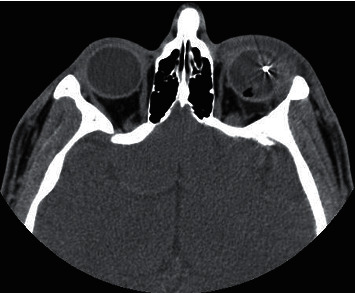
Open globe injury in a 34-year-old man. Axial unenhanced CT image showing a metallic intraocular foreign body.

**Figure 5:**
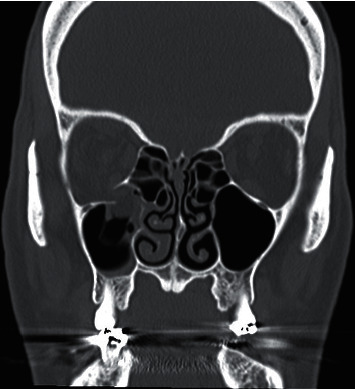
Open globe injury in a 63-year-old man. Axial unenhanced CT image showing concurrent inferior orbital fracture.

**Figure 6:**
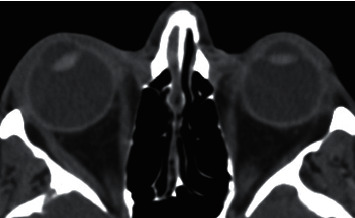
Open globe injury in a 24-year-old man. Axial unenhanced CT image showing anterior chamber deepening and dislocated lens in the right eye.

The findings observed in the CT images of patients with open globe injury were disorganized globe, globe borders haziness, sclera thickening, intraocular foreign body, chamber size increase/decrease, hemorrhage in anterior/posterior chambers, lens dislocation, orbital fracture, hemorrhage, and foreign body (Figures 2–6). Cohen's kappa for interrater agreement was 0.875 and percent of the agreement was 93.846% for CT findings.

### 3.4. Sensitivity, Specificity, Accuracy, and Predictive Values

The prognostic value of the presence of abnormal findings in orbital CT scan for poor final visual acuity in patients with traumatic eye injury using the binary cross tab model is demonstrated in Table 2. The sensitivity (65.96%, 95% CI 57.51%–73.72%), specificity (76.27%, 95% CI 63.41%–86.38%), accuracy (69.00%, 95% CI 62.09%–75.33%), and positive predictive value (86.92%, 95% CI 80.55%–91.42%) showed to be high. However, there was a low negative predictive value (8.39%, 95% CI 41.71%–55.13%) of CT findings for the prediction of poor vision.

### 3.5. Hazard Ratios

The calculated hazard ratios of the presence of each orbital CT scan findings (separately) for predicting poor final visual acuity in patients with traumatic eye injury are presented in Table 3. Among the investigated factors, disorganized/collapsed globe, increased/decreased anterior chamber size, hemorrhage in anterior/posterior chamber, globe borders haziness, orbital foreign body, and optic canal/nerve injury reached the statistical significance for increasing the hazard ratio of poor visual outcome in patients with a traumatic eye injury (for any CT scan finding 6.228, 95% CI 3.112–12.461, *p* < 0.001).

### 3.6. Logistic Regression Analysis

The results of binary logistic regression analysis for detection of independent predictors of poor final visual outcome among orbital CT scan findings are shown in Table 4. Logistic regression analysis showed evidence only for disorganized/collapsed globe (ExpB: 74.327, 95% CI 6.938–796.240, *p* < 0.001) and optic canal/nerve injury (ExpB: 31.605, 95% CI 4.864–205.361, *p* < 0.001) in orbital CT scan as independent predictive factors of poor visual outcome.

## 4. Discussion

In the current study, we have evaluated the predictive value of orbital CT scan findings for poor visual outcomes in patients with a traumatic eye injury. Investigating multiple factors as evidence for globe or orbit injury, such as disorganized or collapsed globe, intraocular foreign body or gas, increased or decreased anterior chamber size, hemorrhage in the anterior or posterior chamber, crystalline or intraocular lens dislocation, posterior sclera thickening, globe borders haziness, orbital fracture, orbital hemorrhage, and foreign body, optic canal, and optic nerve injury, we found that generally, orbital CT findings have an acceptable positive predictive value for poor visual outcome. However, the negative predictive value was not clinically acceptable. This means that the presence of the abovementioned abnormal findings in the orbital CT scan of any patient with traumatic eye injury predicts the poor visual outcome with high accuracy, but the lack of these findings does not rule out the potential poor visual outcome. Among the mentioned factors, only disorganized/collapsed globe, increased/decreased anterior chamber size, hemorrhage in anterior/posterior chamber, globe borders haziness, orbital foreign body, and optic canal/nerve injury reached the statistical significance for increasing the hazard ratio for poor visual outcome in analysis using cross tables. However, in binary logistic regression analysis, the only evidence for disorganized/collapsed globe and optic canal/nerve injury in orbital CT scan remained as independent predictive factors for poor visual outcome.

Multiple studies have evaluated the factors associated with visual outcomes in patients with eye trauma [10, 12–14, 16]. However, no study was found to analyze orbital CT scan clues for this purpose. Rao et al. evaluated multiple clinical factors in predicting the visual outcome of patients with open globe injury [28]. They found the presence of hemorrhage in the anterior or posterior chamber, site and extension of injury, as well as retinal detachment as the significant factors. They also found presenting visual acuity as the only independent predictive factor in regression analysis. Similarly, we found the CT scan clues for hemorrhage in the anterior and posterior chambers as a significant predictive factor for poor visual outcomes. In another survey, by Fujikawa et al., investigating a similar question in 59 Japanese patients found comparable results [12]. Besides the mentioned factors in Rao et al.'s study, they reported the importance of crystalline lens dislocation and history of keratoplasty as predictive factors for visual outcome. In contrast with these findings, the CT scan clues for the dislocation of crystalline lens did not reach statistical significance in our study which may be due to the low number of patients. Two different prognostic models for visual outcome in patients with open globe injury, the classification and regression tree and ocular trauma score, were compared in another research by Man and Steel on 100 patients from the UK [29]. They claimed higher accuracy of ocular trauma score determined by univariate chi-square analysis. This scoring system contains 6 factors based on the first clinical evaluation (presenting vision, presence of globe rupture, perforating injury, endophthalmitis, retinal detachment, and relative afferent pupillary defect) [30]. As we mentioned previously, we skipped these clinical findings and focused on the CT scan findings in our survey because the clinical evaluation of these prognostic factors may not be achievable in many patients with trauma due to their condition.

Our study benefits some strength including novelty in evaluated prognostic factors and a larger sample size compared to the similar mentioned studies. However, it has important limitations in the interpretation of the results. We have indeed focused on orbital CT findings to determine their predictive value. However, in the clinical setting, we cannot rely only on this diagnostic modality for assessing the prognosis. All other achievable clinical findings should be added to our estimation from the patients' visual prognosis and clinical decision-making. It is also important to consider the variability of CT machines as well as imaging protocols for the quality of archived images. The standard slicing wideness for an orbital CT scan is 1-2 mm which is considered a thin cut, while head CT scan is archived with a 10 mm slice in trauma patients [31]. The reference planes used for these two CT scan protocols are also different [31]. So, our results cannot be generalized to the evaluation of orbit by routine head CT scans taken in trauma patients. The experience of radiologists or ophthalmologists reading the orbital CT scan of a patient with traumatic eye injury can also affect the diagnostic accuracy of CT scan findings. So, all of these factors should be considered in the generalizability of our results and applying our findings in clinical practice.

To sum up, our findings showed that CT scan findings can be considered important prognostic factors for visual outcomes in patients with a traumatic eye injury. CT scan findings in these patients have high positive, but limited negative predictive value. Among the findings, evidence for disorganized/collapsed globe and optic canal/nerve injury in orbital CT scan are the most important factors that independently predict the poor visual outcome. Other statistically significant predictive factors are increased/decreased anterior chamber size, hemorrhage in the anterior/posterior chamber, globe borders haziness, and orbital foreign body. The use of these factors for the prediction of visual outcomes in clinical practice should be done with other achievable clinical and physical exam findings and caution regarding the limitations associated with the quality of CT scan machines, the correct applied protocols, and the experience of the interpreter.

## Figures and Tables

**Table 1:** Baseline demographic characteristics of patients with traumatic eye injury in patients with and without CT scan findings.

Characteristics	With CT scan findings Mean ± SD *n* = 93	Without CT scan findings Mean ± SD n = 107	Total Mean ± SD *n* = 200	*P* value^*∗∗*^
Gender (male/female)	71/22	78/29	149/51	0.577

Age (year)	30.78 ± 0.83	29.18 ± 0.84	29.92 ± 0.84	0.185

Education				
Non	24 (%)	35 (%)	59 (29.5%)	0.300
Primary	31 (%)	23 (%)	54 (27%)	
Secondary	28 (%)	36 (%)	64 (32%)	
Tertiary	10 (%)	13 (%)	23 (11.5%)	

Setting of injury				
Recreation	10 (10.8%)	14 (13.1%)	24 (12%)	0.961
Home	24 (25.8%)	30 (28%)	54 (27%)	
Occupation	17 (18.3%)	21 (19.6%)	38 (19%)	
Transportation	6 (6.5%)	6 (5.6%)	12 (6%)	
Intentional	22 (23.7%)	24 (22.4%)	46 (23%)	
Others	14 (15.1%)	12 (11.2%)	26 (13%)	

**Table 2:** Prognostic value of presence of CT scan finding (total) for poor final visual acuity in patients with traumatic eye injury using the binary cross tab model.

Statistic	Value	95% confidence interval
Sensitivity	65.96%	57.51%–73.72%
Specificity	76.27%	63.41%–86.38%
Positive likelihood ratio	2.78	1.73–4.46
Negative likelihood ratio	0.45	0.34–0.58
Positive predictive value	86.92%	80.55%–91.42%
Negative predictive value	48.39%	41.71%–55.13%
Accuracy	69.00%	62.09%–75.33%

**Table 3:** Prognostic value of presence of CT scan findings (separately) for poor final visual acuity in patients with traumatic eye injury using binary hazard ratio calculation.

	Hazard ratio	95% confidence interval		*P* value
		Upper	Lower	
Any CT scan finding	6.228	3.112	12.461	<0.001
Disorganized globe	47.727	6.130	371.628	<0.001
Globe borders haziness	3.063	1.339	7.010	0.006
Posterior sclera thickening	2.577	0.862	7.707	0.081
Intraocular foreign body	1.217	0.434	3.412	0.709
Chamber size increase/decrease	5.043	2.572	9.889	<0.001
Hemorrhage anterior chamber	3.588	1.900	6.774	<0.001
Hemorrhage posterior chamber	3.623	1.905	6.890	<0.001
Lens dislocation	3.171	0.821	12.257	0.079
Orbital fracture	1.854	0.925	3.714	0.079
Orbit hemorrhage	0.388	0.046	3.295	0.369
Orbit foreign body	3.662	1.112	12.051	0.024
Optic nerve/canal injury	21.622	4.733	98.788	<0.001

**Table 4:** Prognostic value of presence of CT scan findings (separately) for poor final visual acuity in patients with traumatic eye injury using binary logistic regression.

	Variables in the equation				95% CI for EXP (B)	
	B	S.E.	Sig.	Exp (B)	Lower	Upper
Disorganized globe	4.308	1.210	0.000	74.327	6.938	796.240
Globe borders haziness	0.924	0.646	0.152	2.520	0.711	8.933
Posterior sclera thickening	−0.592	0.885	0.504	0.553	0.098	3.136
Intraocular foreign body	−1.239	0.832	0.136	0.290	0.057	1.478
Chamber size increase/decrease	1.073	0.574	0.062	2.923	0.950	8.996
Hemorrhage anterior chamber	0.600	0.690	0.384	1.822	0.471	7.046
Hemorrhage posterior chamber	−0.237	0.630	0.707	0.789	0.230	2.712
Lens dislocation	−0.140	1.073	0.896	0.870	0.106	7.126
Orbital fracture	−0.971	0.650	0.136	0.379	0.106	01.355
Orbit hemorrhage	−1.850	1.423	0.194	0.157	0.010	2.557
Orbit foreign body	0.843	0.888	0.342	2.323	0.408	13.237
Optic nerve/canal injury	3.453	0.955	0.000	31.605	4.864	205.361
Constant	−1.744	0.264	0.000	0.175	—	—

## Data Availability

The data used to support this study are available from the corresponding author upon request.
